# DALBACEN cohort: dalbavancin as consolidation therapy in patients with endocarditis and/or bloodstream infection produced by gram-positive cocci

**DOI:** 10.1186/s12941-019-0329-6

**Published:** 2019-10-19

**Authors:** Carmen Hidalgo-Tenorio, David Vinuesa, Antonio Plata, Pilar Martin Dávila, Simona Iftimie, Sergio Sequera, Belén Loeches, Luis Eduardo Lopez-Cortés, Mari Carmen Fariñas, Concepción Fernández-Roldan, Rosario Javier-Martinez, Patricia Muñoz, Maria del Mar Arenas-Miras, Francisco Javier Martínez-Marcos, Jose Maria Miró, Carmen Herrero, Elena Bereciartua, Samantha E. De Jesus, Juan Pasquau

**Affiliations:** 1 Department of Infectious Diseases, Hospital Universitario Virgen de las Nieves, Av. de las Fuerzas Armadas no 2, 18014 Granada, Spain; 2 Hospital Universitario San Cecilio, Granada, Spain; 3 Hospital Regional de Málaga, Málaga, Spain; 4 Hospital Ramón y Cajal, Madrid, Spain; 5 Hospital Universitari Sant Joan, Reus, Spain; 6 Hospital Universitario La Paz, Madrid, Spain; 7 Hospital Universitario Virgen Macarena, Seville, Spain; 8 Hospital Marqués de Valdecillas, Santander, Spain; 9 Hospital Gregorio Marañón, Madrid, Spain; 10 Hospital del Mar, Barcelona, Spain; 11 Hospital Juan Ramón Jiménez, Huelva, Spain; 12 Hospital Clínic, Barcelona, Spain; 13 Complejo Hospitalario de Jaén, Jaén, Spain; 14 Hospital Universitario de Cruces, Bilbao, Spain

**Keywords:** Endocarditis, Bloodstream infection, Dalbavancin

## Abstract

**Objectives:**

To analyse the effectiveness of dalbavancin (DBV) in clinical practice as consolidation therapy in patients with bloodstream infection (BSI) and/or infective endocarditis (IE) produced by gram-positive cocci (GPC), as well as its safety and pharmacoeconomic impact.

**Methods:**

A multicentre, observational and retrospective study was conducted of hospitalised patients with IE and/or BSI produced by GPC who received at least one dose of DBV. Clinical response was assessed during hospitalization, at 3 months and at 1 year.

**Results:**

Eighty-three patients with median age of 73 years were enrolled; 73.5% were male; 59.04% had BSI and 49.04% IE (44.04% prosthetic valve IE, 32.4% native IE, 23.5% pacemaker lead). The most frequently isolated microorganism was *Staphylococcus aureus* in BSI (49%) and coagulase-negative staphylococci in IE (44.1%). All patients with IE were clinically cured in hospital; at 12 months, there was 2.9% loss to follow-up, 8.8% mortality unrelated to IE, and 2.9% therapeutic failure rate. The percentage effectiveness of DBV to treat IE was 96.7%. The clinical cure rate for BSI was 100% during hospital stay and at 3 months; there were no recurrences or deaths during the follow-up. No patient discontinued treatment for adverse events. The saving in hospital stay was 636 days for BSI (315,424.20€) and 557 days for IE (283,187.45€).

**Conclusions:**

DBV is an effective consolidation antibiotic therapy in clinically stabilized patients with IE and/or BSI. It proved to be a cost-effective treatment, reducing the hospital stay, thanks to the pharmacokinetic/pharmacodynamic profile of this drug.

## Background

The incidence of infective endocarditis (IE) has risen over recent decades, attributable to improved patient survival and diagnostic techniques and the increased utilization of invasive devices (e.g., urinary catheters, etc.) and procedures (e.g., haemodialysis, etc.) [1]. IE is most frequently caused by gram positive cocci (GPC), with *Staphylococcus aureus* being the most commonly isolated bacterium. Despite medical and surgical improvements, IE-associated mortality remains high, at around 30% in the first year [2]. Medical treatment of IE requires prolonged periods of parenteral antibiotic therapy that can involve long hospital stays, leading to the development of home antibiotic treatment programmes, now recommended in clinical practice guidelines [3]. Some hospitals with insufficient resources to adopt this approach have implemented outpatient oral antibiotic treatment programmes, with promising results [4].

GPC bloodstream infection is usually associated with the use of a venous catheter and is one of the most frequent nosocomial infections, reported to increase the morbidity and mortality of hospitalised patients by up to 12–25%, augmenting hospital costs [5]. The recommended approach is at least 14 days of antibiotic therapy [6].

Dalbavancin (DBV), an antibiotic from the lipoglycopeptide family, is active against GPCs. Thanks to its pharmacokinetic/pharmacodynamic (PK/PD) characteristics, a single dose of 1000 mg or 1500 mg IV achieves adequate plasma and tissue concentrations to provide antibiotic coverage for 1 or 2 weeks, respectively [7]. However, few clinical data are available on its effectiveness against GPC IE or BSI [8–10].

The objective of this study was to evaluate the effectiveness of DBV in clinical practice as consolidation therapy in patients with GPC IE or BSI and to determine the incidence of adverse effects and its pharmacoeconomic impact and cost-effectiveness.

## Patients and methods

### Study design

We conducted a multicentre, observational, retrospective study (in 14 Spanish hospitals) of hospitalised patients with GPC IE and/or BSI who received at least one dose of DBV prescribed by their attending physician in the clinical setting. The study period ranged from 2016 (when DBV was approved by all participating hospitals) to 31 December 2017. Patients were followed up for 12 months after their first dose of DBV. The study did not involve any direct pharmacological intervention and was approved by the ethics committee of the coordinating hospital, which was also responsible for the quality control of data from the participating hospitals.

### Population

Inclusion criteria were: the presence of IE and/or BSI with microbiological isolation of GPCs, and the receipt of least one dose of DBV after providing written or verbal informed consent to this treatment. Exclusion criteria were: age under 18 years, pregnancy, EI and/or BSI caused by microorganisms other than GPC, moderate/severe liver failure, and/or history of hypersensitivity to glycopeptides.

### Variables

Data were gathered from the clinical history of the patients on the following variables: age and sex of the patient; length of hospital stray (days from date of admission to date of discharge); department at admission and discharge; patient comorbidities (age-adjusted Charlson index); the type of IE for which DBV was prescribed [definite/probable, native/prosthetic, early/late); the device involved (pacemaker, implantable cardioverter-defibrillator); the presence of BSI (complicated, uncomplicated)]; previous and/or concomitant antibiotic therapy for the infection; the microorganism responsible; susceptibility testing results; the date of DBV administration and the dose; the need for surgery in patients with IE and the date; related and unrelated death in hospital or at 12 months; recurrence; reinfection; adverse effects (AEs), including headache, nausea, diarrhoea, vomiting, rash; increased gamma-glutamyl transferase, diarrhoea from *Clostridium difficile*; and DBV discontinuation due to adverse event.

### Definition of variables

Infectious endocarditis (IE) was defined according to 2015 modified Duke criteria [3]. IE was considered early when within 12 months post-surgery and late when more than 12 months post-surgery [11].

- Nosocomial or healthcare-related IE was considered when symptom onset was observed after 48 h of hospital admission or when an invasive procedure had been performed in hospital, nursing home or assisted care facility in the three months before the diagnosis, e.g., intravenous treatment, wound care, or haemodialysis, etc. [12].
- BSI recurrence was defined by the reappearance of the same bacterial strain in blood cultures after completion of treatment [13].
- IE relapse was defined by a second episode of IE due to the same microorganism within three months of the first.
- IE reinfection was defined by another IE during the 12-month follow-up caused by a different microorganism from that identified in the original infection.
- Mortality was evaluated as hospital mortality (death from any cause during hospital stay or the first 30 days post-discharge), and mortality at 3 and 12 months related to IE (e.g., heart failure due to valve dysfunction) and not related to IE (e.g., cancer).
- Microbiological failure was defined by persistent or breakthrough BSI during the treatment of BSI or IE [13], or when the same microorganism was isolated in the blood culture of a patient with IE requiring surgery after completing antibiotic therapy.
- Complicated BSI was defined by the presence of septic metastases and/or the non-removal of a colonised catheter and/or no response at 48–72 h [13].
- The age-adjusted Charlson Comorbidity Index was used to assess the 10-year life expectancy of the patients [14].

### Statistical analysis

The percentage effectiveness was calculated by performing two analyses, one taking account of all patients who received at least one dose of DBV and completed the follow-up and the other also including the patients lost to the follow-up. In a descriptive analysis, absolute and relative frequencies (%) were calculated for qualitative variables, and means and standard deviations for quantitative variables with normal distribution or medians and 25th and 75th percentiles for quantitative variables with non-normal distribution, as established by the Kolmogorov–Smirnov test. p < 0.05 was considered significant. SPSS © 20.0 was used for data analyses.

### Cost analysis

In our evaluation of the economic impact of DBV, its cost was compared with the cost of antibiotics commonly used against GPC IE and BSI, including vancomycin, daptomycin, linezolid, and the cost was then multiplied by the mean number of days of DBV-covered treatment in our patient cohort. We also analysed the savings in hospital costs obtained by using antibiotics for outpatient administration at a day hospital (intravenous daptomycin) or at home (oral linezolid). Doses of antibiotics used for cost analysis were: 1 g vancomycin/12 h (€13.80/day), 700 mg daptomycin/24 h (€140.14/day), 600 mg linezolid/12 h (€71.40/day) and 1500 mg DVB to cover 14 days of treatment (€161.79/day). The cost of 1 day of hospital stay was estimated as €495.95 (according to prices published by the Regional Government of Andalusia).

## Results

### Population description

The study included 83 patients, 73.5% male, with a median age of 73 years (IQR: 53–77 years) and mean Charlson index of 2 (IQR: 1–4). BSI was present in 59.1% of the patients, complicated in 41% and uncomplicated in 18.1%. Definite IE was present in 37.3% of the patients and probable IE in 3.6%.

### Patients with IE

The median age of the 34 patients with IE was 73 years, and 73.5% were male; IE was definite in 91.1% and probable in 8.8%; 44.1% had prosthetic valve IE (29.4% late vs. 14.7% early), 32.4% native valve IE, and 23.5% pacemaker lead IE. IE affected the aortic valve in 50% of these patients, the mitral valve in 23.5%, and the tricuspid valve in 2.5%.

Among the microorganisms, isolated in these patients, 44.1% were coagulase-negative staphylococci (CNS), 29.4% *Staphylococcus aureus* (SA) (20.6% methicillin-susceptible SA (MSSA) and 8.8% methicillin-resistant SA (MRSA), 11.8% streptococci and 8.8% *Enterococcus faecalis*. The reason for DBV administration was the achievement of early discharge in 91.2% of cases and the failure of previous antibiotic therapy in 8.8%. DBV administration reduced the hospital stay by 14 days (IQR: 7–17), producing a total decrease of 557 days. Out of the 17 (50%) patients with IE cases who received surgery, 91.7% received DBV on the next day. The median number of days on antibiotics (68.6% daptomycin, 29.4% ceftriaxone, 23.5% vancomycin and 8.8% linezolid) before DBV administration did not significantly differ between operated and non-operated patients (14.7 vs. 16.8 days, p = 0.529). The median interval between admission and surgery was 8 days (IQR: 6–15). Table 1 exhibits the data for all study variables.

**Table 1:** Characteristics of patients with infective endocarditis

	N = 34
Age, median (IQR)	73 (63–81)
Male, n (%)	25 (73.5)
Charlson index, n (%)	2 (1–4)
Type of infection, n (%)	
Definite IE	31 (91.2)
Probable IE	3 (8.8)
Endocarditis, n (%)	
Native	11 (32.4)
Early prosthetic	5 (14.7)
Late prosthetic	10 (29.4)
Pacemaker lead	8 (23.5)
Valve affected, n (%)	
Aortic	17 (50)
Mitral	8 (23.5)
Tricuspid	1 (2.9)
Causative organism, n (%)	
MSSA	7 (20)
MRSA	3 (8.6)
CNS	15 (42.9)
*E. faecalis*	3 (8.6)
*Streptococcus* spp.	7 (20)
Patient received prior antibiotic therapy, n (%)	34 (100)
Days of previous antibiotic treatment, median (IQR)	28 (17–35)
Prior antibiotic therapy, n (%)	
Daptomycin	24 (68.6)
Ceftriaxone	10 (28.6)
Linezolid	3 (8.6)
Vancomycin	8 (22.9)
Surgery, n (%)	12 (34.3)
Surgery before administering DBV	11 (91.6)
Reason for DBV administration, n (%)	
Facilitate discharge	30 (88.6)
Prior treatment failure	3 (8.6)
DBV dose, n (%)	
1000 mg (1 day), 500 mg (8 days)	10 (29.4)
1000 mg 1 day	5 (14.7)
1500 mg (1 day)	12 (35.3)
1000 mg (1 day), 500 mg (8 days), 500 mg (15 days)	1 (2.9)
1500 mg (1 day), 1000 mg (15 days)	3 (.8)
1500 mg (1 day), 1000 mg (15 days, 30 days, 45 days)	1 (2.9)
1000 mg (1 days), 500 mg every week/9 weeks	1 (2.9)
1500 mg (1 days), 1000 mg every 2 weeks/10 weeks	1 (2.9)
DBV-covered days, median (IQR)	14 (14–21)
Clinical cure, n (%)	34 (100)
Microbiological cure, n (%)	33 (97.1)
Follow-up blood cultures:	17 (48.6)
Negative follow-up blood cultures	17 (100)
IE-related death, n (%)	
During hospitalisation	0
At 12 months	0
Relapse, n (%)	0
Median reduction in hospital stay (IQR)	14 (7–17)
Total reduction, days	557

*MSSA* methicillin-sensitive *S. aureus*; *MRSA* methicillin-resistant *S. aureus*, *CNS* coagulase-negative staphylococcus

### Patients with BSI

Forty-nine patients had GPC BSI, which was complicated in 30.6% of cases; the mean patient age was 67 years and the mean Charlson index was 2 (IQR: 1–4) (see Additional file 1: Table S1). DBV was administered as first option in three patients and after the administration of another antibiotic, for a median of 8 days (IQR: 0–15), in the remainder (93.8%). The reason for DBV prescription was to facilitate hospital discharge in 93.8% of cases. DBV administration achieved a reduction in hospital stay of 14 days (IQR: 7–14). The remaining characteristics of these patients are displayed in Table 2.

**Table 2:** Bloodstream infection characteristics

	N = 49
Age, median (IQR)	67 (50–75)
Male, n (%)	36 (73.5)
Charlson index, n (%)	2 (1–4)
Type of infection, n (%)	
Complicated bloodstream infection	34 (69.4)
Uncomplicated bloodstream infection	15 (30.6)
Causative organism, n (%)	
MSSA	15 (30.6)
MRSA	9 (18.4)
CNS	17 (34.7)
*E. faecalis*	1 (2)
*E. faecium*	2 (4.1)
Streptococcus	2 (4.1)
Other	1 (2)
No isolation	2 (4.1)
Days of prior AB therapy, median (IQR)	8 (0–15)
Prior antibiotic therapy, n (%)	46 (93.9)
Daptomycin	22 (44.9)
Ceftriaxone	10 (20.4)
Linezolid	9 (18.4)
Vancomycin	11 (22.4)
Reason for DBV administration, n (%)	
Facilitate discharge	38 (77.6)
Prior treatment failure	1 (2)
Toxicity	2 (4)
DBV dose, n (%)	
1000 mg (1 day), 500 mg (8 days)	14 (28.6)
1000 mg 1 day	11 (22.4)
1500 mg 1 day	21 (42.9)
Other	3 (6.1)
DBV-covered treatment days (IQR)	14 (14)
Clinical cure, n (%)	49 (100)
Microbiological cure	
Follow-up blood cultures, n (%)	36 (73.5)
Negative follow-up blood cultures	35 (97.2)
Death related to bloodstream infection, n (%)	0
Relapse, n (%)	0
Readmission for different reason	1 (2)
Reduction in days of hospital stay (IQR)	14 (7–14)
Total reduction, days	636

### Effectiveness

#### IE

Of the 34 patients with IE, 15 (44.1%) had prosthetic valve IE, which was treated with surgery in 10 (66.7%) of these cases and resolved with antibiotic therapy alone in the remaining 5 (33.5%). Out of the 8 (23.5%) with pacemaker lead IE, 7 (87.5%) were cured with antibiotic therapy and removal of the system; the remaining patient (12.5%) was considered a failure, being initially diagnosed with pacemaker pocket infection due to *S. epidermidis* and treated by removal of the generator together with antibiotic therapy (daptomycin 10 mg/kg/24 h/IV + gentamycin 5 mg/kg/24 h/IV + rifampicin 600 mg/24 h/IV for 2 weeks, and a single dose of dalbavancin 1500 mg/IV); at one month, a haematoma was detected in the pocket of the extracted pacemaker, the leads were removed, and *S. epidermidis* was again isolated. Out of the 11 (32.5%) patients with native valve endocarditis, 5 (45.4%) required surgery and the remaining 6 were cured with antibiotic therapy alone (Table 3).

**Table 3:** Outcomes of infective endocarditis

	IE type	Micro-organism	Reason for surgery	Before-DBV treatment (weeks)	Reasons for DBV prescription	Adverse effects	Cure of infection	DBV dose (mg)
1	LPVE	MSSA	Yes (1)	1st. Vancomycin (< 1), and cloxacillin (2); 2nd. surgery; 3rd. cloxacillin (2) plus fosfomycin (2)	Outpatient management	No	Yes	1000 (1 day) 500 (8 days)
2	EPVE	*S. epidermidis*	Yes (1)	1st. Daptomycin (< 1) plus rifampicin (< 1); 2nd. vancomycin (2) plus rifampin (2); 3rd. surgery; 4th. vancomycin plus rifampicin (2)	Outpatient management	No	Yes	1000 (1 day) 500 (8 days)
3	EPVE	MRSA		1st. Vancomycin (< 1); 2nd. daptomycin (3) plus fosfomycin (3)	Outpatient management	Yes (fever)	Yes	1000 (1 day) 500 (8 days) 500 (15 days)
4	PLE	*S. epidermidis*	Yes (2)	1st. Amoxicillin-clavulanic acid (< 1); 2nd. linezolid (3); 3rd. surgery; 4th. daptomycin (4)	Outpatient management	No	Yes	1500 (1 day)
5	NVE	S. agalactiae	Yes (3)	1st. Vancomycin (< 1); 2nd. penicillin G (3) plus gentamycin (3); 3rd. surgery; 4th. ceftriaxone (1)	Outpatient management	No	Yes	1500 (1)
6	LPVE	*S. epidermidis*.		1st. Moxifloxacin (< 1); 2nd. daptomycin (1), cloxacillin (1) plus gentamycin (1), plus rifampicin (1); 3rd. daptomycin (6)	Outpatient management	No	Yes Death not-related with IE (6 months later)	1000 (1 day) 500 (8 days)
7	NVE	MSSA		1st. Daptomycin (1) plus gentamycin (1); 2nd. cloxacillin (3) plus gentamycin (1)	Outpatient management	No	Loss to follow up	1000(1 day)
8	NVE	Unknown		1st. Ceftriaxone (< 1); 2nd daptomycin (< 1); 3rd. imipenem/cilastatin (1)	Outpatient management	No	Yes	1500 (1 day) 1000 (15 days)
9	LPVE	*S. epidermidis*.		1st. Cloxacillin (< 1) plus daptomycin (< 1); 2nd. daptomycin (3) plus gentamycin (2) plus fosfomycin (2)	Previous treatment failure	No	Yes	1500 (1 day)
10	PLE	*S. epidermidis*.	Yes (4)	1st. Daptomycin (1), gentamycin (1), rifampicin (1), 2nd. surgery	Outpatient management	Yes (renal failure)	Treatment failure	1500 (1 day)
11	NVE	*S. agalactiae*		1st. Ertapenem (< 1); 2nd. ampicillin (1) plus gentamycin (1); 3rd. ceftriaxone (1)	Outpatient management	No	Yes	1500 (1 day) 500 (8 days)
12	NVE	*E. faecalis*		1st. Meropenem (< 1); 2nd. ampicillin (3) plus ceftriaxone (3); 3rd. linezolid (1); 4th. daptomycin (2)	Outpatient management	No	Yes	1000 (1 day)
13	NVE	*S. lugdunensis*	Yes (1)	1st. Cloxacillin (3); 2nd. surgery; 3rd daptomycin (1)	Outpatient management	No	Yes	1500 (1 day)
14	EPVE	CNS	Yes (1)	1st. Aztreonam (< 1); 2nd. daptomycin (2) plus rifampicin (2) plus fosfomycin (2); 3rd. surgery; 4th. daptomycin (2) plus rifampicin (4) plus fosfomycin (4)	Outpatient management	No	Yes	1500 (1 day)
15	LPVE	MSSA		1st. Piperacillin/tazobactam (1); 2nd. cloxacillin (1) plus daptomycin (1) plus rifampicin (1); 3rd. cloxacillin (2) plus rifampicin (2) plus fosfomycin (< 1)	Outpatient management	No	Yes	1000 (1 day)
16	NVE	*S. viridans*		1st. Amoxicillin-clavulanate (1); 2nd. ceftriaxone (3)	Outpatient management	No	Yes	1500 (1 day)
17	PLE	*S. epidermidis*	Yes (4)	1st. Cloxacillin (< 1) plus gentamycin (< 1) plus rifampicin (1); 2nd. surgery; 3rd. daptomycin (2)	Outpatient management	No	Yes	1500 (1 day)
18	PLE	*S. epidermidis*	Yes (4)	1st. Rifampicin (1), gentamycin (1), daptomycin (1); 2nd. surgery	Outpatient management	No	Yes	1500 (1 day)
19	PLE	*S. schleiferi*	Yes (4)	1st. Ceftriaxone (< 1); 2nd. surgery; 3rd. cloxacillin (2)	Outpatient management	No	Yes	1000 (1 day) 500 (8 days)
20	PLE	*S. epidermidis*	Yes (4)	1st. Meropenem (< 1); 2nd. surgery; 3rd. daptomycin (2)	Outpatient management	No	Yes	1000 (1 day)
21	NVE	MSSA	Yes (1)	1st. Ceftriaxone (< 1), 2nd. daptomycin (< 1) plus gentamycin (1); 3rd. cloxacillin (1); 4th. Surgery; 5th. cloxacillin (2) plus rifampicin (1)	Outpatient management	No	Yes	1500 (1 day)
22	EPVE	MRSA	Yes (1)	1st. Daptomycin (2), gentamycin (2), rifampicin (2); 2nd. surgery; 3rd daptomycin (1)	Previous treatment failure	No	Yes	1500 (1 day) 1000 (15 days)
23	NVE	*E. faecalis*		1st. Ampicillin plus ceftriaxone (4)	Outpatient management	No	Yes	1000 (1) 500 (8 days)
24	NVE	MSSA		1st. Ceftriaxone (< 1) plus levofloxacin (< 1); 2nd. cloxacillin (1); 3rd. cefazolin (1)	Outpatient management	No	Yes Death not-related with IE (3 months later)	1000 (1 day)
25	PLE	CNS	Yes (5)	1st. Daptomycin (1), gentamycin (1), rifampicin (1); 2nd. surgery; 3rd. daptomycin (1)	Outpatient management	No	Yes	1000 (1 day) 5002 (8 days)
26	PLE	CNS	Yes (6)	1st. Levofloxacin (2); 2nd. daptomycin (2); 3rd. surgery; 4th. daptomycin (2)	Outpatient management	No	Yes	1000 (1 day) 500 (8 days)
27	LPVE	MSSA		1st. Vancomycin (< 1) plus gentamycin (< 1) plus rifampin (< 1), 2nd. daptomycin (1) plus rifampicin (1); 3rd. cloxacillin (2) plus rifampicin (2)	Outpatient management	No	Yes	1000 (1 day) 500 (8 days)
28	LPVE	MRSA		1st. Vancomycin (2), 2nd. linezolid (1); 3rd. ceftaroline (4)	Previous treatment failure	No	Yes	1000 (1 day) 500 mg/every week/9 weeks
29	LPVE	*S. bovis*		1st. Vancomycin (< 1) plus rifampicin (< 1) plus gentamycin (< 1), 2nd. ceftriaxone (4) plus gentamycin (4)	Outpatient management	No	Yes Death not-related with IE (4 months later)	1500 (1 day) 1000/every 2 weeks/10 weeks
30	LPVE	*S. lugdunensis*		1st ceftriaxone (< 1); 2nd cloxacillin (2)	Outpatient management	No	Yes	1000 (1 day) 500 (8 days)
31	NVE	MR *S. epidermidis*		1st. Daptomycin (4)	Outpatient management	No	Yes	1000 (1 day) 500 (8 days)
32	EPVE	MSSA		1st. Levofloxacin (< 1), 2nd. daptomycin (< 1), 3rd. cloxacillin (4) plus rifampicin (3) plus gentamycin (2)	Outpatient management	No	Yes	1500 (1 day)
33	LPVE	*E. faecalis*	Yes (1)	1st. Vancomycin (2), gentamycin (< 1); 2nd. surgery; 3rd. vancomycin (1)	Outpatient management	No	Yes	1500 (1 day) 1000 (15 days) 1000 (30 days) 1000 (45 days)
34	LPVE	*S. epidermidis*	Yes (1)	1st. Daptomycin (2) plus rifampicin (2); 2nd. surgery; 3rd. daptomycin (2)	Outpatient management	No	Yes	1500 (1 day)

MSSA: methicillin-sensitive *S. aureus*; MRSA: methicillin-resistant S*. aureus*; CNS: coagulase negative staphylococcus; *LPVE* late prosthetic valve endocarditis; *NVE* native valve endocarditis; *EPVE* early prosthetic valve endocarditis; *PLE* pacemaker lead endocarditis

(1) Aortic or mitral valve replacement. (2) Electrode removal. (3) Mitral valve repair with ring. (4) Electrode and pacemaker lead removal. (5) Pacemaker removal and pus drainage from pocket. (6) Pacemaker lead removal without removing electrode from the implantable automatic defibrillator

All patients with IE were clinically cured during their hospital stay and there was only one microbiological failure (in the patient with pacemaker lead IE).

The 12-month follow-up was completed by 33 (97.1%) of the patients with EI, with only one loss (due to the patient leaving the country). Three (9.1%) died for causes unrelated to DBV treatment: one for adenocarcinoma of the oesophagus, one for decompensated liver cirrhosis, and the third for a new episode of native IE at four, six and three months after hospital discharge, respectively. Two (6.1%) patients had a new episode of endocarditis: one had pacemaker lead endocarditis caused by *Serratia* spp at 10 months after the first episode of PLE by *S. epidermidis,* which was treated by complete removal of the pacemaker system and antibiotics; and the other patient had native endocarditis due to MSSA at three months after the first NVE episode by a different microorganism, who was treated with DBV and met the clinical, microbiological and echocardiographic criteria for a cure.

At 12 months, the proportion of patients cured of IE was 85.3% when the patient lost to the follow-up was included and 96.7% when only patients completing the one-year follow-up were considered. There was no relapse at 12 months.

#### BSI

A clinical cure was obtained in all of the 49 patients with GPC BSI, and post-treatment blood cultures ordered for 36 (73.5%) of the patients were all negative. There were no relapses at 90 days. One patient was readmitted for BSI *S. epidermidis* and developed fever 1 month later, when he was diagnosed with pacemaker lead endocarditis due to Corynebacterium.

### Adverse events

Among the 83 patients who received DBV, 4.8% experienced adverse effects: asthenia, self-limited rash, fever with self-limited shivering, and impaired renal function that was found by laboratory tests to have returned to its baseline status at one week. There was no grade 4 event or any event causing treatment discontinuation, and no patient developed colitis due to *Clostridium difficile.*

### Pharmacoeconomic study

The DBV dose administered was designed to achieve a mean antibiotic coverage of 2 weeks. The cost of 2 weeks of treatment with the antibiotics under study was 193.20€ for vancomycin, 1960.20€ for daptomycin, 999.60€ for linezolid and 2265.00€ for DBV.

The mean reduction of 14 days in the hospital stay of DBV-treated patients represented an estimated saving of 6938.26€. The total cost of 14 days of antibiotic therapy, including the cost of hospital stay, was 7131.46€ for vancomycin, 8898.46€ for daptomycin, 999.6€ for linezolid and 2265€ for dalbavancin.

The total reduction in hospital stay in the present cohort was 636 days for patients with BSI (315,424.20€) and 557 days for those with IE (283,187.45€). Treatment with DBV allowed the early discharge of 71 patients (84.5%) with an overall saving of 1193 days (mean of 14 days/patient, range 7–14).

## Discussion

This cohort of 83 elderly patients with endovascular infection by GPC were predominantly male, and two in five had IE. Older patients are frequently polymedicated, increasing the possibility of therapeutic failure due to poor treatment compliance [15] and the risk of drug interactions [16]. The pharmacokinetic/pharmacodynamic properties of DBV mean that a single dose can provide antibiotic coverage for 1 or 2 weeks, favouring compliance to the treatment regimen, and this drug does not interact with cytochrome p450, reducing the likelihood of potential drug interactions [17].

A clinical cure rate of 100% was achieved in both BSI and IE when DBV was used as a consolidation strategy in this study population, with a microbiological cure rate of 100% in BSI and 97.1% in IE. Furthermore, no relapse or BSI- or IE-related death was recorded during the hospital stay or at 3 or 12 months post-discharge. In two-thirds of the patients with prosthetic IE, surgery was not required, and DBV was administered as consolidation therapy to facilitate discharge.

The incidence of BSI has increased over recent decades, with no major improvements in its prognosis, and it is the seventh leading cause of death in Europe and North America [18]. IE is a severe disease mostly caused by GPC, with *S. aureus* being the most frequently implicated microorganism. It commonly affects older individuals and is associated with a high mortality rate despite a correct treatment [19]. DBV is a lipoglycopeptide approved for the treatment of skin and soft tissue infections. It was found to be more effective than vancomycin or linezolid to treat GPC BSI (100% vs. 85.7%) [18], and the only clinical trial on its utilisation.

against GPC BSI also found a higher cure rate with DBV (87%) than with vancomycin (50%) [8]. In addition, a Spanish retrospective study on the administration of DBV in routine clinical practice observed a cure rate of 84.1% in patients with BSI secondary to catheter infection [9]. There has been only one single-centre retrospective study on the effectiveness of DBV in GPC IE, which followed 27 patients for 2 years and reported a cure rate of 92.6% [10]. Out of the 7 (25.9%) patients with prosthetic valve IE, this was resolved by the antibiotic therapy alone in six and was treated by surgery in the remaining patient, whose IE was produced by *Enterococcus faecalis* and who died 2 weeks post-surgery; out of the with device-related IE, a cure was achieved by surgery in all cases except for one patient whose device could not be completely removed [10].

To date, the treatment of choice for patients with prosthetic valve or medical device IE has been the combination of antibiotic therapy with valve replacement or removal of the complete system whenever possible [3]. In vitro studies on biofilm-associated infections reported that DBV is efficacious against prosthetic joint infections produced by staphylococci [20] or vancomycin-susceptible Enterococci [21] and against catheter infections caused by MRSA and *S. epidermidis* [22]. These findings may explain the excellent outcomes achieved by DBV in our study, in which around half of the patients with prosthetic IE were cured with no need to remove the prosthetic material.

In the present study of DBV, the drug-related adverse event rate was below 5%, and there were no grade 4 events or events prompting treatment discontinuation; in addition, no *Clostridium difficile* strains were detected. In one clinical trial, a rate of 12.3% was obtained, with a severe adverse event rate of only 0.3% [23], and another trial observed an adverse event rate below 5%, including nausea, diarrhoea, elevated LDH, headache, rash, and vomiting [24]. The duration of antibiotic treatment against IE ranges between 4 and 8 weeks, usually leading to changes in intestinal microbiota and increasing the risk of adverse events, including *C. difficile* colitis. However, in comparison to other antibiotics habitually used to treat IE, these changes and the possibility of *C. difficile* infection are minimized when DBV is administered, in part explaining the low adverse event rate observed with this drug [25].

DBV proved to be a cost-effective antibiotic in our study population. It was generally used as an antibiotic consolidation strategy, facilitating discharge, reducing stay, and maximising treatment adherence. The mean hospital stay is considered to be 7–14 days for BSI [18] and 4–8 weeks for IE, depending on the microorganism involved, the valve affected, the need for surgery, and associated complications [26]. Our results indicate that the selection of DBV can reduce the hospital stay of patients with complicated or uncomplicated BSI caused by DBV-susceptible GPCs and of patients with IE who do not require surgery or who are stable and complication-free post-surgery and only require hospitalization for the intravenous antibiotic administration. DBV has previously been described as a cost-effective antibiotic against GPC infections, including BSI and osteoarticular infections [27], and against skin and soft tissue infections caused by MRSA [28].

The limitations of our study include its retrospective, observational, non-interventional design and the lack of a comparator antibiotic. The strength of our study lies in the collaboration of 14 hospitals with considerable experience in endovascular infection, yielding the largest sample to date of patients with GPC IE and/or BSI for analysis of the effectiveness of DBV in real-life clinical practice.

DBV appears to be effective against GPC IE and BSI as consolidation antibiotic therapy in patients who are already clinically stabilized. Besides the important clinical advantages of DBV, including increased treatment adherence, its PK/PD profile offers major pharmacoeconomic benefits, allowing the hospital stay to be significantly reduced.

## Supplementary information

**Additional file 1: Table S1.** Comorbidities of patients with endocarditis and bloodstream infections.

## Data Availability

The researchers confirm the accuracy of the data provided for the study and its availability.

## References

[1] Van den Brink FS, Swaans MJ, Hoogendijk MG, Alipour A, Kelder JC, Jaarsma W (2017). European Society of Cardiology guideline update: a nationwide study in the Netherlands. Eur Heart J Qual Care Clin Outcomes..

[2] Cahill TJ, Baddour LM, Habib G, Salaun E, Pettersson GB (2017). Challenges in infective endocarditis. J Am Coll Cardiol..

[3] Habib G, Lancellotti P, Antunes MJ, Bongiorni MG, Casalta JP, Del Zotti F (2015). 2015 ESC Guidelines for the management of infective endocarditis: The Task Force for the Management of Infective Endocarditis of the European Society of Cardiology (ESC). Endorsed by: European Association for Cardio-Thoracic Surgery (EACTS), the European Association of Nuclear Medicine (EANM). Eur Heart J..

[4] Iversen K, Ihlemann N, Gill SU, Madsen T, Elming H, Jensen KT (2019). Partial oral versus intravenous antibiotic treatment of endocarditis. N Engl J Med..

[5] Ferrer C, Almirante B (2014). Infecciones relacionadas con el uso de catéteres vasculares. EIMC..

[6] Chong YP, Moon SM, Bang KM, Park HJ, Park SY, Kim MN (2013). Treatment duration for uncomplicated *Staphylococcus aureus* bacteremia to prevent relapse: analysis of a prospective observational cohort study. Antimicrob Agents Chemother.

[7] Koulenti D, Xu E, Mok IYS, Song A, Karageorgopoulos DE, Armaganidis A, Lipman J, Tsiodras S (2019). Novel antibiotics for multidrug-resistant gram-positive microorganisms. Microorganisms..

[8] Raad I, Darouiche R, Vazquez J, Lentnek A, Hachem R, Hanna H (2005). Efficacy and safety of weekly dalbavancin therapy for catheter-related bloodstream infection caused by gram-positve pathogens. Clin Infect Dis..

[9] Bouza E, Valerio M, Soriano A, Morata L, Carus EG, Rodríguez-González C (2018). Dalbavancin in the treatment of different gram-positive infections: a real-life experience. Int J Antimicrob Agents.

[10] Tobudic S, Forstner C, Burgmann H, Lagler H, Ramharter M, Steininger C (2018). Dalbavancin as primary and sequential treatment for Gram-positive infective endocarditis: 2-year experience at the general hospital of Vienna). Clin Infect Dis.

[11] Almirante B, Miró JM (2008). Infections associated with prosthetic heart valves, vascular prostheses, and cardiac pacemakers and defibrillators. Enferm Infecc Microbiol Clin.

[12] Ben-Ami R, Giladi M, Carmeli Y, Orni-Was-serlauf R, Siegman-Igra Y (2004). Hospital-acquired infective endocarditis: should the definition be broadened?. Clin Infect Dis.

[13] Cisneros-Herreros JM, Cobo-Reinoso J, Pujol-Rojo M, Rodríguez-Baño J, Salavert-Lletí M (2007). Guidelines for the diagnosis and treatment of patients with bacteriemia. Guidelines of the Sociedad Española de Enfermedades Infecciosas y Microbiología Clínica. Enfer Infecc Microbiol Clin.

[14] Charlson ME, Charlson RE, Paterson JC (2008). The Charlson comorbidity index is adapted to predict costs of chronic disease in primary care patients. J Clin Epidemiol.

[15] Milton JC, Hill-Smith I, Jackson SH (2008). Prescribing for older people. BMJ.

[16] Mallet A, Spinewine A, Huang A (2007). The challenge of managing drug interactions in elderly people. Lancet..

[17] Dash RP, Babu RJ, Srinivas NR (2017). Review of the pharmacokinetics of dalbavancin, a recently approved lipoglycopeptide antibiotic. Infect Dis.

[18] Nilesen SL (2015). The incidence and prognosis of patients with bacteremia. Dan Med. J.

[19] Alkhawam H, Sogomonian R, Zaiem F, Vyas N, El-Hunjul M, Jolly J (2016). Morbidity and mortality of infective endocarditis in a hospital system in New York City serving a diverse urban population. J Investig Med..

[20] Fernández J, Greenwood-Quaintance KE, Patel R (2016). In vitro activity of dalbavancin against biofilms of staphylococci isolated from prosthetic joint infections. Diagn Microbiol Infect Dis.

[21] Neudorfer K, Schmidt-Malan SM, Patel R (2018). Dalbavancin is active in vitro against biofilms formed by dalbavancin-susceptible enterococci. Diagn Microbiol Infect Dis.

[22] Díaz-Ruíz C, Alonso B, Cercenado E, Cruces R, Bouza E, Muñoz P, Guembe M (2018). Can dalbavancin be used as a catheter lock solution?. J Med Microbiol..

[23] Boucher HW, Wilcox M, Talbot GH, Puttagunta S, Das AF, Dunne MW (2014). Once-weekly dalbavancin versus daily conventional therapy for skin infection. N Engl J Med.

[24] Jauregui LE, Babazadeh S, Seltzer E, Goldberg L, Krievins D, Frederick M (2005). Randomized, double-blind comparison of once-weekly dalbavancin versus twice-daily linezolid therapy for the treatment of complicated skin and skin structure infections. Clin Infect Dis.

[25] Nord CE, Rasmanis G, Wahlund E (2006). Effect of dalbavancin on the normal intestinal microflora. J Antimicrob Chemother.

[26] Wang A, Gaca JG, Chu VH (2018). Management considerations in infective endocarditis: a review. JAMA.

[27] Morrisette T, Miller MA, Montague BT, Barber GR, Mc Queen RB, Krask M (2019). On- and off-label utilization of dalbavancin and oritavancin for Gram-positive infections. J Antimicrob Chemother.

[28] Agarwal R, Bartsch SM, Kelly BJ, Prewitt M, Liu Y, Chen Y, Umscheid CA (2018). Newer glycopeptide antibiotics for treatment of complicated skin and soft tissue infections: systematic review, network meta-analysis and cost analysis. Clin Microbiol Infect.

