# Supplementary material for: DALBACEN cohort: dalbavancin as consolidation therapy in patients with endocarditis and/or bloodstream infection produced by gram-positive cocci
Source: Ann Clin Microbiol Antimicrob. 2019 Oct 19;18:30. doi: 10.1186/s12941-019-0329-6 (PMC6800500; doi:10.1186/s12941-019-0329-6)
Supplement: Supplementary file 1 — Additional file 1: Table S1. Comorbidities of patients with endocarditis and bloodstream infections. [file 12941_2019_329_MOESM1_ESM.doc]

***Additional file 1: Table S1. Comorbidities of patients with endocarditis and bloodstream infections***

|  | **Bloodstream infection n=49** | **Endocarditis n=34** |
| --- | --- | --- |
| **Charlson index** | 2 (1-4) | 2 (1-4) |
| **Heart disease** | 26 (53.1) | 28 (82.3) |
| **Chronic renal failure** | 12 (24.5) | 3 (8.8) |
| **Respiratory disease** | 9 (18.4) | 7 (20.5) |
| **Diabetes mellitus** | 15 (30.6) | 10 (29.4) |
| **Neurological disease** | 5 (10.2) | 2 (5.8) |
| **Immunosuppressants** | 5 (10.2) | 0 |
| **Corticosteroids** | 10 (20.4) | 1 (2.9) |
| **HIV** | 1 (2) | 0 |
| **Solid organ transplant** | 2 (4.1) | 0 |
| **Haematological transplant** | 0 | 0 |
| **Surgery in previous 3 months** | 5 (10.2) | 5 (14.7) |
| **Gastrointestinal disease** | 6 (12.2) | 3 (8.8) |
| **Active neoplasm** | 7 (14.3) | 0 |
| **Chemotherapy** | 3 (6.1) | 0 |
| **Liver disease** | 5 (10.2) | 3 (8.8) |

HIV: human immunodeficiency virus infection.
